# Evidence That Primary Visual Cortex Is Required for Image, Orientation, and Motion Discrimination by Rats

**DOI:** 10.1371/journal.pone.0056543

**Published:** 2013-02-18

**Authors:** Sarah K. Petruno, Robert E. Clark, Pamela Reinagel

**Affiliations:** 1 Division of Biological Sciences, University of California San Diego, La Jolla, California, United States of America; 2 Veterans Affairs Medical Center, University of California San Diego, La Jolla, California, United States of America; Universität Bielefeld, Germany

## Abstract

The pigmented Long-Evans rat has proven to be an excellent subject for studying visually guided behavior including quantitative visual psychophysics. This observation, together with its experimental accessibility and its close homology to the mouse, has made it an attractive model system in which to dissect the thalamic and cortical circuits underlying visual perception. Given that visually guided behavior in the absence of primary visual cortex has been described in the literature, however, it is an empirical question whether specific visual behaviors will depend on primary visual cortex in the rat. Here we tested the effects of cortical lesions on performance of two-alternative forced-choice visual discriminations by Long-Evans rats. We present data from one highly informative subject that learned several visual tasks and then received a bilateral lesion ablating >90% of primary visual cortex. After the lesion, this subject had a profound and persistent deficit in complex image discrimination, orientation discrimination, and full-field optic flow motion discrimination, compared with both pre-lesion performance and sham-lesion controls. Performance was intact, however, on another visual two-alternative forced-choice task that required approaching a salient visual target. A second highly informative subject learned several visual tasks prior to receiving a lesion ablating >90% of medial extrastriate cortex. This subject showed no impairment on any of the four task categories. Taken together, our data provide evidence that these image, orientation, and motion discrimination tasks require primary visual cortex in the Long-Evans rat, whereas approaching a salient visual target does not.

## Introduction

Primary visual cortex (V1) is one of the most studied and best understood areas of the mammalian brain, and has served as a model for understanding cortex generally. Much is known about the local circuitry and visually evoked activity within V1, the anatomy and physiological response properties of its thalamic inputs, and the visual information contained in V1 projections to downstream targets. This raises the exciting possibility of accounting quantitatively and mechanistically for visual behaviors in animals using highly constrained computational models incorporating exhaustive functional and structural data. Rodent models are important to this effort because of the accessibility to a broad range of tools [1], [2]. Their small size facilitates anatomic tracing, EM reconstruction, and optical imaging. The ease of genetic manipulation makes them ideal for use of genetically encoded sensors and effectors to monitor or manipulate activity in specific cell types, and to map their morphology and connectivity. Quantitative visual behavior paradigms are now available for rats [3], [4] and mice [4], [5], [6], [7], [8]. Multiple extrastriate visual areas have been described in the rat [9], [10] and mouse [10], [11], [12], consistent with hierarchical processing comparable to the primate visual system.

An important goal of rodent vision research is to explain visual behavior in terms of V1 structure and function. For this program to be fruitful it will be important to verify which visual behaviors rely obligatorily on V1 in rodents. The question arises because human patients with damage to V1 have spared visually guided behaviors, despite being subjectively blind in the affected visual field. The possibility of V1-independent visual responses was first recognized when it was observed that cortically blind patients could make visually guided saccades to unseen targets in their blind field [13], and exhibited pupil dilation reflexes to such targets [14]. The term ‘blindsight’ was originally coined to describe these automatic orienting responses.

Cortically blind patients could also make accurate visually guided voluntary reaching movements to targets in their blind field [15], [16]. The visual behaviors that were spared in cortically blind fields were not found for stimuli in the blind spot caused by the optic disc [17], ruling out the interpretation that spared cortex or scattered light explain all cases of blindsight [18]. Performance in detection of light stimuli in the blind field depended critically on response modality: performance was at chance for verbal report, but high if subjects responded by blinking or pointing [19], [20], suggesting that different motor response modalities have more or less access to the information in V1-independent visual pathways.

Sensitivity to movement of objects in the cortically blind visual field is also well documented in human patients [21], [22], [23]. At least rough discrimination of motion direction in the absence of conscious perception of motion has been reported in some patients [21], [22]. Optic flow processing to compute ego motion is also reported to be intact [24], which may explain intact ability to navigate cluttered environments in blindsight patients. A few patients even report conscious visual awareness of motion stimuli in the affected visual field, despite severe V1 lesions and cortical blindness with respect to other stimuli [25].

In some cases, cortically blind human patients retain rudimentary form discrimination for stimuli in the blind field. When present, form discrimination is inaccessible by verbal report, but well above chance for guided actions. For example, reaching movements were reported to have hand postures appropriate for grasping an object the size and shape of unseen targets [20], [26]. When asked to “post a letter” in an oriented slot presented in the blind field, a cortically blind patient made appropriate hand rotations to match the letter to the orientation of the slot, despite protesting that he could not see any slot and performing at chance on verbal guessing of its orientation [26]. Another patient was able to perform above chance at visually discriminating among four shapes (X, O, vertical line, horizontal line) in his blind field, though he was unable to make same-different judgments between two stimuli presented in the blind field simultaneously [15]. Together these data suggest that form discrimination can be supported through a V1-independent pathway, at least for low level local features.

Many of these V1-independent visually guided actions are attributed to the Superior Colliculus (SC), a sub-cortical structure that receives direct retinotopic input from retinal ganglion cells, and which plays a central role in orienting behaviors [15], [27], [28]. The SC was directly shown to be involved in these spared visual abilities in V1-lesioned primates [29], [30]. The SC sends information to extrastriate visual cortex by way of the pulvinar, bypassing V1. This model of blindsight is posed within the framework of the two streams hypothesis, which holds that extrastriate visual processing is split into independent dorsal (action) and ventral (perception) streams [31]. In primates, only the dorsal stream receives collicular input, which would explain why visual guidance of action (but not visual perception) is spared.

The SC is thought to underlie motion discrimination without V1 as well. Neurons in SC are strongly driven by motion stimuli. Motion-selective neurons in extrastriate cortex (MT) receive input from the SC by way of the pulvinar. The selectivity of MT responses to direction of motion is intact and SC-dependent after V1 lesions in macaque monkeys [32], [33]. In the absence of the SC, however, motion tuning in MT is also intact, as long as V1 is present. Discrimination of direction of motion in blindsight is dependent on dorsal extrastriate cortex in human patients [34], and specifically requires MT [35]. Thus motion processing in the dorsal stream can be supported by either a tectofugal (SC-derived, V1-independent) or a thalamocortical pathway.

While the SC is known to respond to motion stimuli and to have a key role in guiding orienting movements including saccades and reaching, it is usually not implicated in form vision. It has been speculated that orientation and shape discrimination in blindsight could in principle be dependent on the SC by way of connections between dorsal and ventral extrastriate cortex [36]. The recent discovery of direct projections from the dLGN to extrastriate cortex [37], however, presents a second possible substrate for spared form discrimination as well as other aspects of blindsight. There is recent evidence supporting an essential role for the dLGN in some blindsight behaviors [38].

A more exhaustive discussion of the neuroanatomy of human blindsight in can be found elsewhere [39], [40], [41]. For the present study the important conclusion is that there is precedent for V1-independent visually guided action in a wide range of tasks, including not only detection and motion discrimination but also potentially orientation and shape discrimination. There is no precedent, however, for discrimination of complex feature conjunctions or visual object recognition without V1.

The case in rats has been controversial. Lashley reported that V1 is required for visual behavior in rats [42]. But then several studies reported V1-independent visual behaviors. For example, an avoidance-based visual detection task was found to be spared after V1 lesion; V1-independent detection did not require extrastriate cortex, but did require the SC [43], [44]. Other studies reported that completely decorticated rats could acquire and perform a 2-alternative forced choice visual discrimination of grating orientation working for food or liquid rewards [45], [46]. These and other reports of V1-independent visual behavior [47], [48], [49], [50] were countered by other studies that found V1-dependence [51], [52], [53], [54], [55], [56], [57], [58].

Task details, including motor response modality, may be crucial determinants of which V1-independent visual discriminations are revealed in rats [59], as is the case for humans [19], [20]. This might account for at least some of the apparently conflicting findings in the literature. All the earlier experiments with rats used classical behavioral paradigms that differ substantially from the computer-generated CRT-displays, licking response modality, and water reward that we and others now use to study vision in rats and mice. Therefore a re-evaluation in this new context is required.

We study vision in Long-Evans rats because of their demonstrated ability to learn and reliably perform complex visual tasks [3], [8], [60], [61], [62], [63]. Our ultimate goal is to link visual behavior to neural encoding of visual stimuli in identified neural populations. Therefore we tested the V1-dependence of four visual tasks: Approach Salient Visual Target, Motion Discrimination, Orientation Discrimination, and Image Discrimination.

## Results

We trained Long-Evans rats to perform multiple 2AFC visual tasks. In each task, the subject initiated a trial by licking a centrally positioned sensor, at which time a visual stimulus appeared and persisted until a response was made. A response consisted of licking either the left or right response port. Correct responses were rewarded with water, whereas incorrect responses were penalized with a time-out before another trial could be initiated. Four task categories were used in this study: Approach Salient Visual Target, Image Discrimination, Motion Discrimination, and Orientation Discrimination.

**Figure 1:**
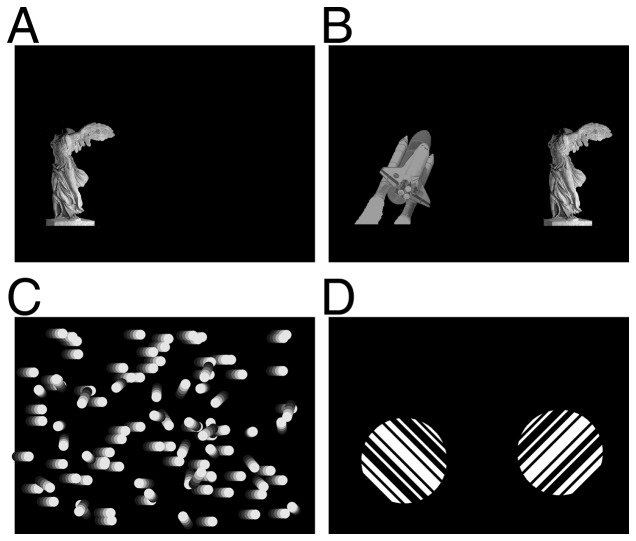
Visual Tasks. **A.** Schematic of stimulus for Approach Salient Visual Target task. Subjects were rewarded for licking the water port beneath the image of the statue. **B.** Schematic of stimulus for the Image Discrimination task. Subjects were rewarded for licking the port beneath the statue, not the space shuttle. **C.** Schematic of stimulus for Motion Discrimination task. Subjects were rewarded for licking the port on the side toward which the coherent dots moved. **D.** Schematic of stimulus for orientation discrimination task. Subjects were rewarded for licking the port beneath clockwise rotated bars, not counter-clockwise rotated bars.

In the *Approach Salient Visual Target* task (Figure 1A) a grayscale photograph of a real-world object (a statue) appeared on one side of the screen, immediately above one of the response ports. Subjects were rewarded for responding on the side where the image appeared. We designed this task to be easily solved by a simple salience computation, as the target images were both brighter and higher contrast than the background (black screen). We anticipated spared behavior in this task after V1 lesion, which would serve as an internal control for the intact status of numerous other aspects of behavior in the event of deficits in other visual tasks.

In the *Image Discrimination* task (Figure 1B) two grayscale images appear, one above each response port. Subjects were rewarded for licking the port on the side with the image they had previously learned to approach in the first task (the statue), and not the port under the distractor image (the space shuttle). We required the rats to discriminate these images independent of their orientation and size. There is no precedent in the literature for V1-independent complex form recognition. We anticipated impairment after V1 lesion, which would serve as a control for our V1 lesion in the event that other visual behaviors are spared.

The *Motion Discrimination* task (Figure 1C) required discriminating the direction (left or right) of a highly salient full-field optic flow stimulus. Rats were rewarded for licking the response port on the side towards which the coherent dots were drifting. The human literature suggests that this task might be V1-independent, but the case for rats was unknown.

The *Orientation Discrimination* task (Figure 1D) required discriminating clockwise (CW) from counterclockwise (CCW) rotated gratings presented in disc apertures. Orientation tuning is the first-described and best-studied property of V1 neurons [64] and is widely presumed to be V1-dependent. Although rodents lack topographical orientation maps in V1, the individual neurons are known to be sharply tuned for orientation [65], [66]. This task is a strong candidate for a V1-dependent behavior. Given that spared orientation discrimination behavior has been reported in both humans and rats, however, it was uncertain whether this specific task would be V1-dependent in rodents.

### Subject 1: Effect of a V1 Lesion

**Figure 2:**
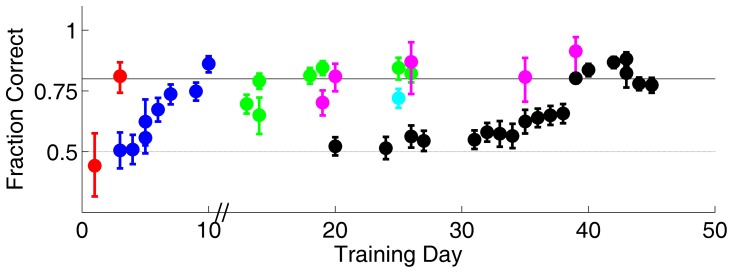
Training on multiple visual tasks: subject S1. Each symbol shows the fraction of correct responses for one task at the end of one day. Color indicates task as follows: Approach Salient Visual Target (Figure 1A) with statue target (red) or CW grating target (magenta); Image Discrimination (Figure 1B) (blue); Motion Discrimination (Figure 1C) with 85% coherent motion (green); and Orientation Discrimination (Figure 1D). Error bars indicate the 95% binomial confidence bounds. Training day indicates calendar days, except that a three week gap in training occurred after day 10 (slash marks). Performance is only reported when the rat's side bias was <15% and ≤40 valid trials completed on a given day; training occurred on other days and tasks not meeting these criteria.

The performance during training for one subject (S1) is shown in Figure 2. This subject acquired all four tasks in about two months: approaching a visual target, discriminating visual images, discriminating direction of motion, and discriminating orientation.

**Figure 3:**
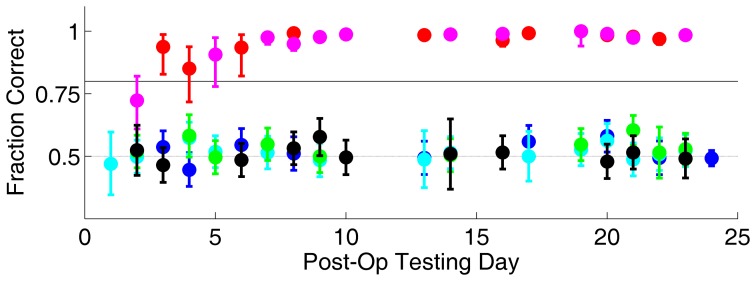
Performance on visual tasks after V1 lesion. Post-operative performance of subject S1, whose pre-operative performance is shown in Figure 2. Symbols and error bars as defined in Figure 2. In addition, motion discrimination was tested with 95% coherent motion (cyan) post-lesion. Performance fell to chance for motion discrimination tasks (cyan, green), image discrimination (blue) and orientation discrimination (black), yet was spared for the tasks requiring approaching a salient visual target (red, magenta).

After training, this subject received a permanent lesion of V1 by stereotaxic injection of ibotenic acid (see Methods). Three identically trained control subjects received sham surgeries. Following a 7 day recovery, the previously learned visual behaviors were re-tested for S1 (Figure 3) as well as sham controls (described below).

Performance on the Image Discrimination task fell to chance after the V1 lesion (51% correct, p<0.05 binomial confidence interval 49–53%). This constitutes a severe deficit compared with pre-lesion performance (86% correct, confidence interval 83–89%) or sham controls (N = 3, 85±4% correct, mean±SD). Performance showed no recovery over 24 days of post-operative testing.

The Approach Salient Visual Target behavior was completely spared, however (98% correct, binomial confidence interval 97–99%), and not different from sham controls (N = 3, 97.1±0.2% correct). Performance on the approach task improved relative to pre-lesion (81% correct, confidence interval 74–87%); similar improvement was also found in sham controls (see below). This spared behavior serves as an internal control that peripheral sensory, cognitive, memory, motivational, and motor systems were intact in subject S1 after the V1 lesion, at least to the extent required for behavior in this paradigm.

Performance on the Motion Discrimination task also fell to chance after the V1 lesion (53% correct, confidence interval 51–56%), a severe deficit relative to either pre-lesion performance (82% correct, confidence interval 81–84%) or sham controls (N = 2; 82±2% correct). The subject S1 showed no recovery of motion discrimination over the testing period, even when we tested a motion stimulus that is easier for intact rats (slower motion and higher coherence).

Performance on the Orientation Discrimination task also fell to chance after the V1 lesion (51% correct, confidence interval 49–53%), a severe deficit compared to pre-lesion performance (82% correct, confidence interval 81–83%). None of the sham control rats had learned the orientation task prior to the surgery date, but see subject S2 below. Performance on this task did not recover over the testing period.

**Figure 4:**
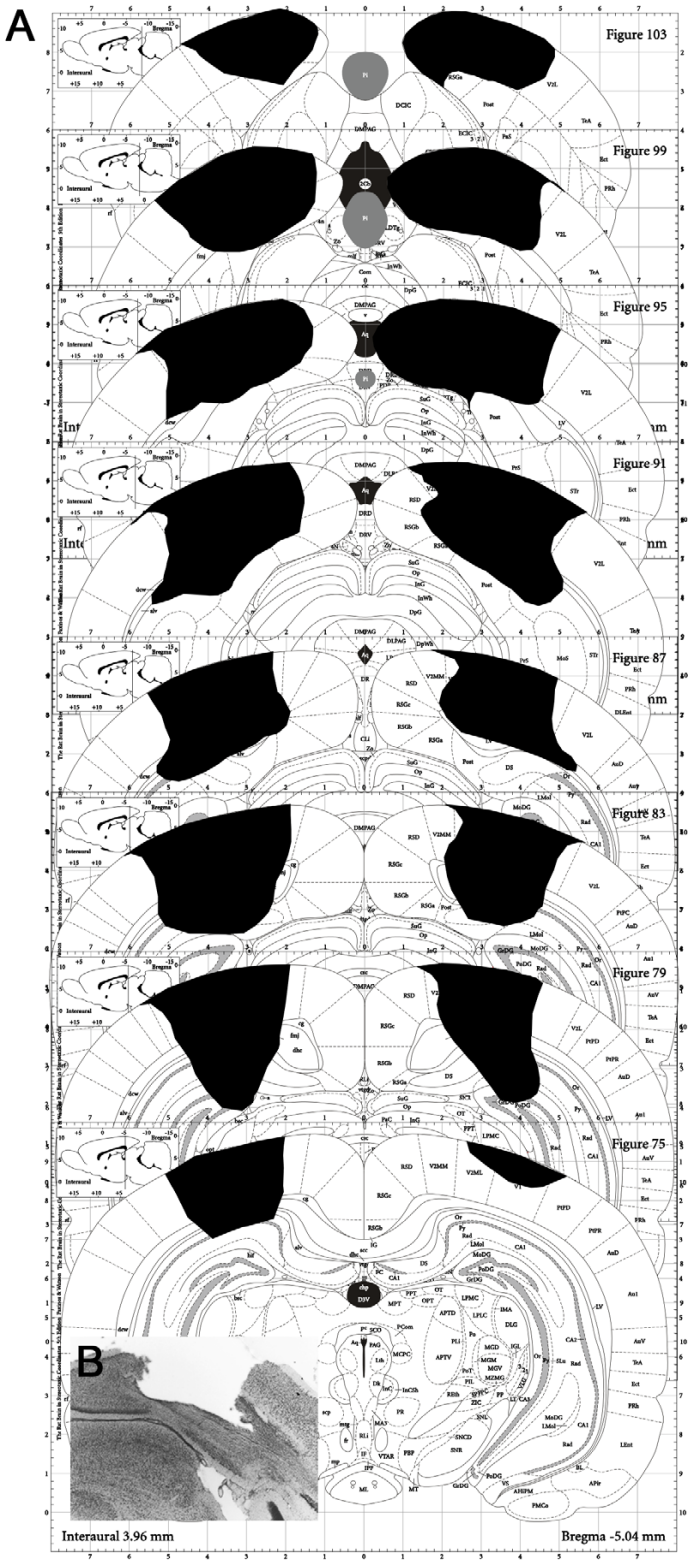
Lesion boundaries for subject S1. **A.** Summary of lesion boundaries. This subject had extensive bilateral damage to area V1. The estimated Right Side % V1 damage was 97%. The estimated Left Side % V1 damage was 84%. The only sparing of V1 tissue was at the most extreme lateral edges that formed the border of V2 and the sparing was slightly more extensive on the left side. Some minor sparing also occurred to the thin lateral band of V1 and extends most anterior. Extra V1 damage occurred to V2 that borders the medial aspect of V1 and the retrosplenial cortex. Minor damage also occurred to the post subiculum and the dentate gyrus. **B.** Sample section through V1 illustrating quality and extent of the damage attained by ibotenic acid injection.

In summary, subject S1 had a profound and persistent deficit in image discrimination, motion discrimination, and orientation discrimination, with complete sparing of a visual approach behavior. This subject had a precise and complete bilateral lesion of V1 (97% of R V1 and 84% of L-V1), as subsequently verified by histology (Figure 4).

### Subject 2: Effect of a lesion to extrastriate visual cortex

**Figure 5:**
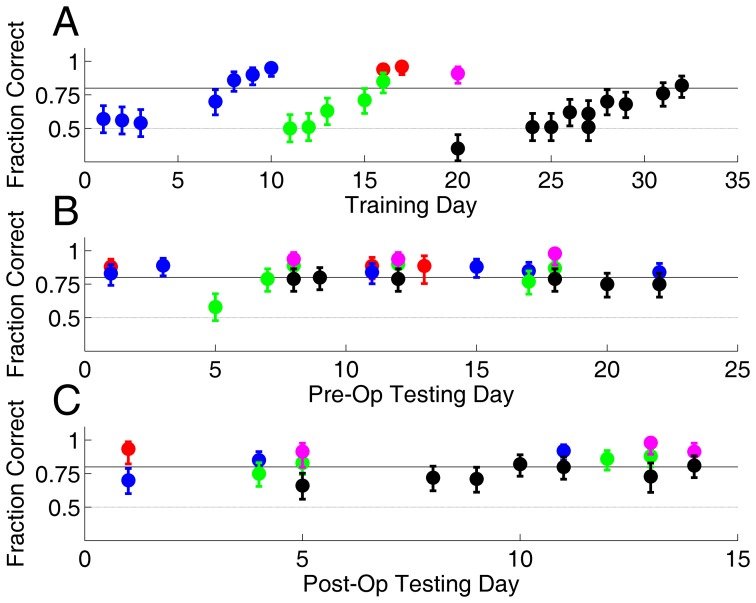
Performance on visual tasks: subject S2. Symbol colors and error bars as defined in Figure 2. **A.** Task acquisition. This subject had learned an Approach Visual Target task previously (not shown). **B.** Stability of performance on visual tasks immediately after acquisition and before surgery. **C.** Post-operative performance after medial extrastriate lesion. No behavioral deficits were observed for approaching salient visual targets (red, magenta), image discrimination (blue), random dot motion discrimination (green), or orientation discrimination (black).

**Figure 6:**
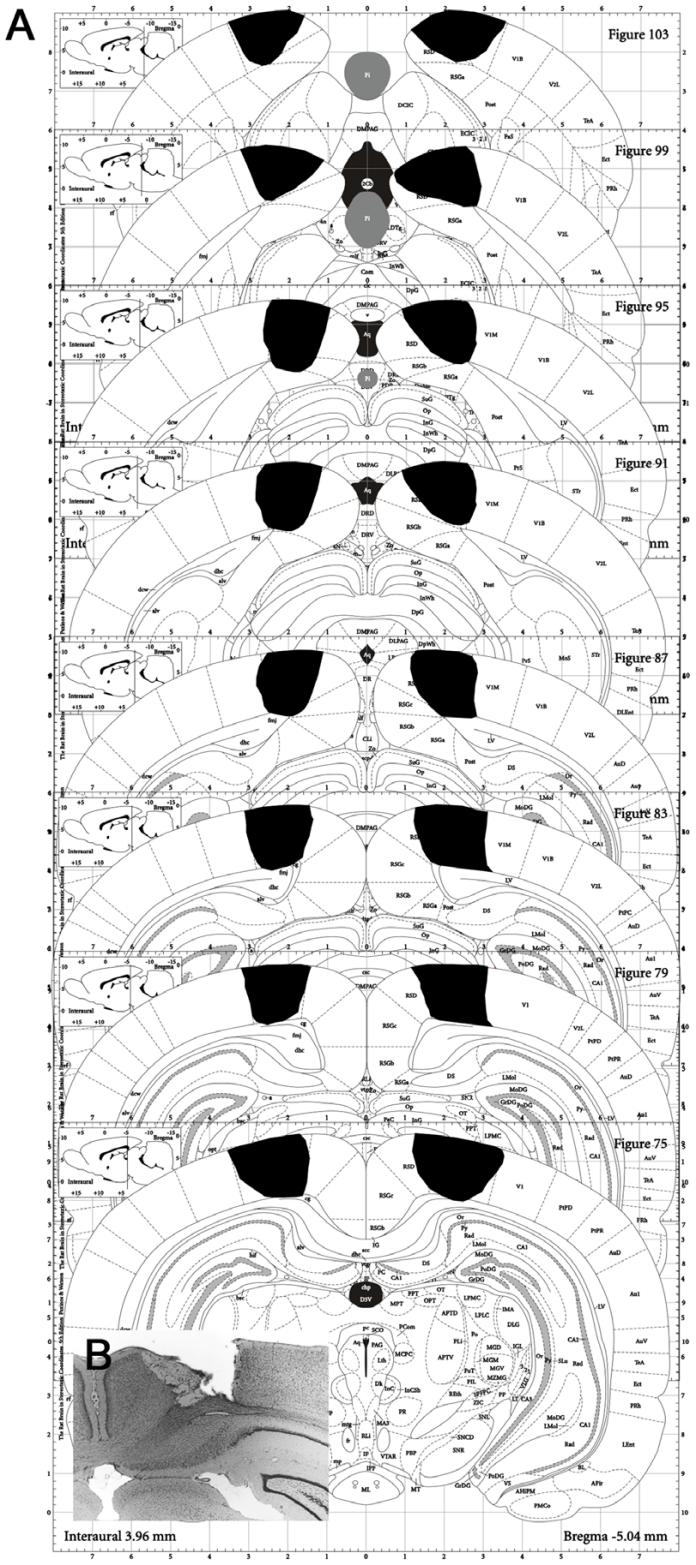
Lesion boundaries for subject S2. **A.** Summary of lesion boundaries. Medial extrastriate visual cortex (areas V2-MM and V2-ML as defined by (Paxinos and Watson, 1998)) was almost completely damaged and the damage was bilaterally symmetrical. There was bilateral sparing in the most anterior sections; the lesion began bilaterally at the same level in V2MM, sparing the most anterior tip of V2-ML bilaterally. Moving posterior, the lesion completely encompassed V2MM and V2ML bilaterally. Overall, 93% of V2MM was damaged and 88% of V2ML was damaged, bilaterally. The extent of V1 damage was mainly to the medial edge of V1. However, at the most posterior portion of the lesion, the V1 damage was more substantial as V1 moves more medially in this region to replace V2MM. Nonetheless, the V1 damage was less than 5% in total. V2-L (the portion that is lateral to V1) was never damaged. **B.** Sample section through lesion illustrating quality and extent of the damage attained by ibotenic acid injection.

A second lesion subject (S2) also learned all four visual tasks (Figure 5A). Stability of performance on all tasks was assessed for an additional 15 days after training and before surgery (Figure 5B). The subject then received a bilateral lesion to the visual extrastriate cortex that lies medial to V1, comprising areas designated V2-ML and V2-MM by standard atlases [67]. After a 7-day recovery period behavioral performance was re-tested. All the visual behaviors that were impaired by V1 lesion (Figure 3) were completely spared after this extrastriate lesion (Figure 5C). Subsequent histology showed that the lesion spared V1 (<5% damage), but ablated 88% of V2-ML and 93% of V2-MM bilaterally (Figure 6).

### Population Summary

**Figure 7:**
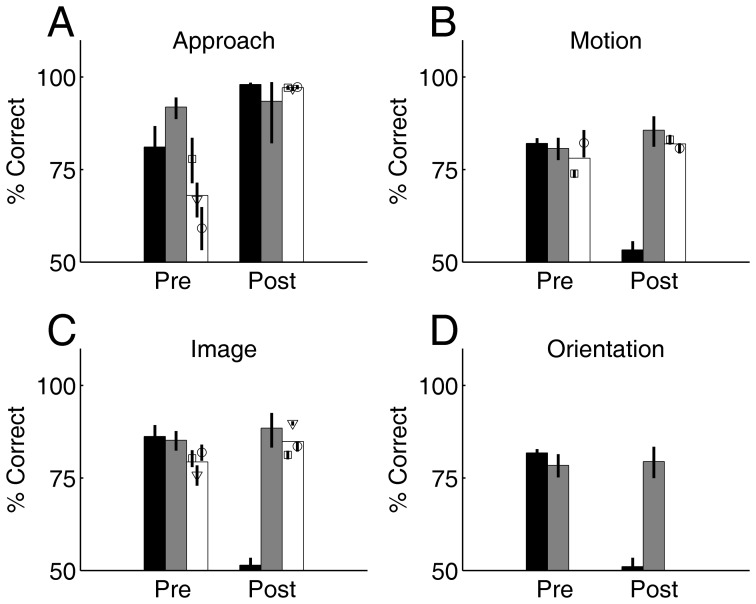
Summary of population results. Each panel shows pre- and post-lesion performance for V1 lesion (subject S1, black bar), medial extrastriate lesion (subject S2, gray bar), and the average of the sham lesion controls (S3–5, white bar), as well as the individual sham control subjects (superimposed symbols). Error bars indicate 95% binomial confidence intervals. **A.** Approach Salient Visual Target task (statue target). **B.** Motion Discrimination task, 85% coherent motion. **C.** Image Discrimination task. **D.** Orientation Discrimination task, which was learned before surgery by only two subjects, S1 and S2. The V1 lesion subject had large and highly significant deficits in motion, image, and orientation discrimination tasks, whether compared to its own pre-lesion performance, compared to sham or extrastriate lesion performance, or compared to post-lesion performance of the same subject on the approach task.

The pre- and post- operative performance of the V1 lesion, medial extrastriate lesion, and three sham controls are summarized in Figure 7. Performance on the Approach Salient Visual Target task was unaffected by the lesions in either V1 or medial extrastriate cortex (Figure 7A). We attribute the post-operative improvement on the task to practice: the V1 lesion and sham controls had only been trained to criterion (not asymptotic performance) before surgery. For subject S2 we trained to asymptotic performance before lesion (Figure 5B), and no further improvement was found post-operatively on the approach task.

The Image Discrimination task was severely impaired after V1 lesion relative to either pre-operative performance or sham controls (Figure 7B). This task was unaffected by the medial extrastriate lesion. Similarly, performance in the Motion Discrimination was abolished by the V1 lesion, completely spared after the extrastriate lesion, and unchanged in sham controls (Figure 7C). Only the two lesion subjects (S1 and S2) learned the orientation discrimination task; performance was severely impaired by the V1 lesion and unaffected by the medial extrastriate lesion (Figure 7D).

## Discussion

We have tested the effects of a V1 lesion on visual behavior in pigmented rats. In one highly informative subject, a complete and precise lesion of area V1 resulted in an absolute and persistent deficit in image discrimination, motion discrimination, and orientation discrimination, while completely sparing performance on approach to visual targets. Given the possibility of redundant pathways or post-surgical recovery, it is remarkable to find such clear, complete, selective and stable deficits. We cannot rule out that damage to adjacent structures contributed to these deficits, but the extent of damage outside V1 in this subject was slight.

The deficits were confirmed by numerous internal and parallel controls: relative to pre-lesion performance (within subject, within task, across surgical status); relative to the spared approach task (within subject, within surgical status, across task); relative to sham lesion controls (within surgical status, within task, across subject). An additional control was provided by the lack of any deficits on the same tasks in a second subject that received a lesion to medial extrastriate visual cortex, sparing V1. Taken together, our data are consistent with the interpretation that V1 is required for image discrimination, random dot motion discrimination, and orientation discrimination, but dispensable for approaching salient visual targets.

Our data suggest that the medial extrastriate cortex is not required for any of these tasks, but the interpretation of this is will depend on the still-evolving definitions of rat extrastriate cortical areas. While earlier studies defined the large area of visual cortex postsynaptic to V1 collectively “V2”, it was later appreciated that the rat has multiple extrastriate cortical areas, each with its own retinotopic map [9] and projection patterns [11], [68]. In the mouse these areas have been found shown to have distinct visual response properties [10], [12]. Our lesion removed the extrastriate visual cortex medial to V1, which is denoted V2-ML and V2-MM in the atlas [67]. This corresponds to areas AM (anteromedial) and PM (posteromedial) [9], [10] or MX_a_ and MX_p_ (medial circumstriate, anterior and posterior) [68]. Our lesion spared the extrastriate visual cortex lateral to V1, denoted V2-L in the atlas [67]. The spared region contains at least four additional distinct extrastriate cortical areas [9], [10], [68], most likely including the rat homolog of primate V2 [11], [68].

Because orientation tunin is the predominant feature of V1 neurons, orientation discrimination and orientation tuning have dominated most studies of V1 in the rodent [5], [12], [65], [69], [70], [71], [72], [73], [74], [75]. Moreover, the mechanisms underlying orientation tuning in V1 have been studied extensively in other species and have been modeled in some detail. This makes orientation discrimination an ideal candidate for intensive structure/function analysis using the powerful tools available in rodent preparations. Our data establish conditions under which orientation discrimination is V1-dependent in the rat; thalamocortical anatomy and physiology should be relevant to explaining behavioral performance on this task. We note however that we used small, high spatial frequency gratings. Our data do not exclude the possibility that large or low-frequency gratings may be behaviorally discriminable by rats in the absence of V1 [43], [44].

Approaching a salient visual target was V1-independent, however. Therefore when behavioral tasks are used to study V1 function, it will be important to take care to avoid inadvertent salience cues that could be used to solve the task. In this context we note that our image discrimination task used invariance to size and rotation to disrupt any local cues or inadvertent salience cues that might have distinguished the images even after normalization of low-order image statistics. Our orientation discrimination task used oblique gratings with matched vertical and horizontal components to avoid potential artifacts that can otherwise arise due to the horizontal scan path of the monitor.

Approaching salient visual targets would clearly be a poor task for studying visual computations in V1. The robustness of this behavior, however, makes it an excellent control task. This task would also be ideal behavioral test for function in the retina. All retinal ganglion cell classes in the rat send a collateral projection to the SC, where we speculate the visual computations underlying the approach behavior likely occur. We are not aware of any evidence for a projection from the dLGN to extrastriate visual cortex in the rat, but the possible existence and participation of such projections must also be considered.

The random dot motion task is widely used in human and primate vision research, and has begun to be used in rodents as well [76]. Given that this stimulus is effective at driving cells in the SC, and discrimination is commonly spared in human patients with V1 lesions, this could easily have been a V1-independent behavior in rats. Our results show, however, that this motion discrimination behavior is V1-dependent in the rat, at least in the context of the specific task we tested.

Our image discrimination task was designed to be especially demanding, requiring invariance to rotation and size, so it is not surprising that V1 was required for the task. We have not tested whether simpler visual form discrimination tasks would be V1-independent, but the orientation discrimination deficit we observed suggests that this is unlikely. We had previously shown that a related image discrimination task does not require perirhinal cortex, a cortical area which lies downstream of the visual processing hierarchy [63]. The lack of any deficit after ablating the medial areas of extrastriate cortex (Figure 5) suggests that one or more of the lateral extrastriate targets of V1 [68], [77] likely participate in image discrimination.

The evidence we presented for a role of V1 in visual behavior is based on an individual case study. Additional subjects will be required to verify the universality of the findings and to refine the boundaries of the cortical areas required for these tasks. Additional task parameters will need to be explored to determine the range of conditions under which these conclusions hold. Nevertheless this individual case is unusually clear and informative, and the data may be immediately useful to the field. Much of the modern visual neuroscience research program is aimed at explaining visual behaviors based on the physiology and anatomy of primary visual cortex, with increasing dependence on rodent models. Our data contribute to this program by providing evidence that the thalamocortical pathway is required in rats for several canonical visual behaviors.

## Methods

### Ethics

All procedures were conducted with care to avoid any pain or suffering in animal subjects. This work was conducted in an AAALAC-accredited facility with the approval and under the supervision of the Institutional Animal Care and Use Committee at the University of California San Diego.

### Selection of subjects

We present here quantification of pre- and post-operative behavior for five rats: N = 1 rat with >90% lesion of V1; N = 1 rat with >90% lesion of V2 ML/MM; and N = 3 rats with sham lesions. Other subjects were excluded because they either failed to learn tasks (N = 8) or had unsuccessful lesions as judged by histology (N = 4).

### Visual training and testing methods

Training procedures and apparatus were essentially as described in [3]. We trained male Long-Evans rats (Harlan) from age P30 in a transparent Lucite training chamber with a CRT visual display on one wall. The CRT monitor (NEC FE992-19, 100 Hz, 1024×768 resolution) was linearized with a minimum, mean, and maximum luminance of 4, 42, and 80 cd/m^2^, respectively (Colorvision, spyder2express). From the position of the center request port, the monitor was about 10 cm from the rat's eye and subtended 104° of visual angle (0.1 degrees/pixel).

Along the bottom of the display were arrayed three response/reward ports which detected licks and dispensed small volumes of liquid reward (water or dilute saccharine water). All tasks had a self-paced, two-alternative forced-choice (2AFC) structure. Subjects initiated a trial voluntarily by licking the center port, which triggered the appearance of a visual stimulus that provided information about the location of reward. The visual stimulus persisted indefinitely until the subject licked either the correct port (in which case the reward was received), or the incorrect port (in which case a time-out occurred). During a time-out the apparatus was dark, silent, and unresponsive; return of mean gray luminance to the monitor indicated to the rat that a new trial could be initiated. Under these conditions, subjects completed most trials within 2s, performed between 200 and 2000 trials per 2-hour training session, and took from 3 days to 3 weeks to acquire each visual task. Subjects moved automatically through a sequence of shaping steps specifying a number of visual tasks as described in Results. On tasks for which stimulus parameters were varied, the values were chosen independently each trial and uniformly over the interval indicated. These parameter statistics were the same for all rats and fixed for the duration of the experiment.

In the first training step the trial-initiating central lick was also rewarded (20–50% the volume of correct response rewards), and incorrect answers were not penalized (time-out = 1 ms). Once subjects performed 200 trials (regardless of performance, usually within 1–3 days) they graduated to the standard reinforcement parameters, in which center lick request was unrewarded, and incorrect responses were penalized with a 2s time-out (increased to up to 8s as needed on an individual basis). In all training steps, correction trials followed 20% of incorrect responses. In correction trials, a new visual stimulus was selected randomly as for a new trial, but the stimulus was presented such that the correct answer was assigned to the same response side as the previous (failed) trial. This was effective at overcoming side bias as well as perseveration over long periods of training, but induced a measurable alternation bias after wrong answers. Therefore only trials following correct answers were included in analysis.

Subjects trained for 2 hours/day, 7 days/wk and earned 100% of their daily water in task performance while maintaining normal weight and growth; supplemental water was given only on days when training was skipped. The operant chamber was cage-attached, such that subjects had free access to food during (as well as between) training sessions; simultaneous access to food and water may be a factor in successful weight maintenance without supplemental water.

Preliminary studies showed that once these tasks are learned to criterion in this paradigm, performance is stable even without practice: subjects retest at the same performance level within a day or two of resuming testing, after breaks of up to 6 months without visual testing or training (data not shown).

### Task and stimulus details

In the *Approach Salient Visual Target* task subjects were rewarded for responding on the side where the image appeared, as described in text. We varied the size (50–100%) and rotation (−30° to +30°) of the image randomly from trial to trial. Most subjects acquire this task within a few days of training, and generalized immediately to other visual targets.

In the *Image Discrimination* task subjects were rewarded for licking the port on the side with the target image (*Nike of Samothrace* statue) and not the side with the distractor image (NASA space shuttle), as described in text. The source images were matched for size (area in pixels^2^) and normalized for luminance and contrast. At full-size, the images filled about half the monitor height (40 degrees visual angle). The sizes and rotations of the two images were independently varied from trial to trial, in the same manner described for the previous task. This means the rats must approach the statue for reward even in trials when the space shuttle is the larger and brighter of the two stimuli, making it unlikely that an Approach Salient Target strategy was employed. Rats which failed to acquire the second task after three weeks of training were removed from the study (see Selection of Subjects above).

The *Motion Discrimination* task was a full-field random dot coherent motion task. One hundred white dots, each 1.8 degrees visual angle in diameter, appeared against a black background at random locations across the entire display; 85% of these drifted coherently either to the left or to the right at a speed of 60 degrees per second. The remaining dots moved in random directions. The stimulus continued indefinitely until subjects responded; as dots moved off screen they were replaced with new dots starting at random locations. We previously determined these parameters to be easy for Long-Evans rats to discriminate once trained [76]. In addition, subject S1 was tested with 95% coherence, 30 degrees per second motion after the lesion (Figure 3, cyan).

The *Orientation Discrimination* task required discriminating clockwise (CW) from counterclockwise (CCW) rotated gratings presented in disc apertures, over small perturbations in orientation and size. The gratings were 100% contrast square wave gratings with stripes of random width (white noise in one dimension). The rewarded orientation was the same for all rats (CW) and cued by pre-training the rats on Approach to Salient Visual Target using only the target orientation as a target. The same randomly generated grating patch was rotated CW (+40° to +50°) and CCW (−40° to −50°) to create a discrimination pair. The rotations of the target and distractor gratings were varied from trial to trial independently of one another over the indicated range. The sizes of the two grating patches were yoked within a trial, but varied slightly from trial to trial (75–100%). At 100% size the grating patch was 36 degrees visual angle in diameter. The gratings contained spatial frequencies ranging from 0.2–5 cycles/degree. This spans the range previously determined to be above threshold for Long-Evans rats (data not shown). Orientation was the only stimulus feature predictive of reward location in this task.

### Lesion surgery

Anesthesia was maintained throughout surgery with isoflurane gas (0.8%–2.0% isoflurane delivered in O2 at 1 L/min). The rat was placed in a Kopf stereotaxic instrument, and the incisor bar was adjusted until Bregma was level with Lambda. Bilateral excitotoxic perirhinal lesions were produced by local microinjections of ibotenate acid (IBO; Biosearch Technologies, San Rafael, CA). IBO was dissolved in 0.1 M phosphate-buffered saline to provide a solution with a concentration of 10 mg/ml, pH 7.4. A volume of 0.1 to 0.35 ul was injected at a rate of 0.1 µl/min with a 10 µl Hamilton syringe mounted on a stereotaxic frame and held with a Kopf Microinjector (model 5000). The syringe needle was lowered to the target coordinate and left in place for 1 min before beginning the injection. Following the injection, the syringe needle was left in place for a further 2 min to reduce the spread of IBO up the needle tract. In subject S1 we injected 0.35 ul IBO at a depth of 1.2–1.5 mm from the brain surface at each site. There were seven injection sites in each hemisphere evenly spaced in the area defined by 5.8–9.3 mm AP, 2.2–4.2 mm ML relative to Bregma, bilaterally. In subject S2 we injected 0.2 ul IBO at a depth of 1.0–1.2 mm from the brain surface at each site. There were six injection sites in each hemisphere evenly spaced in the area defined by 4.3–7.8 mm AP, 1.1–3.4 mm ML relative to Bregma, bilaterally. Once awake and responsive, each rat was returned to its home cage in the colony room for a 7-day recovery period. The procedure for the sham-operated control group was the same as for the lesion groups, with the exception that the dura was not punctured, the syringe needle was not lowered into the cortex, and no IBO was injected.

### Histology and lesion quantification

At completion of testing, the rats were administered an overdose of sodium pentobarbital and perfused transcardially with buffered 0.9% NaCl solution followed by 10% formaldehyde solution (in 0.1 M phosphate buffer). The brains were then removed and cryoprotected in 20% glycerol/10% formaldehyde. Coronal sections (50 mm) were cut with a freezing microtome beginning at the level of the anterior commissure and continuing caudally until then entire posterior neocortical area had been sliced. Every fifth section was mounted and stained with thionin to assess the extent of the lesions. Lesion size estimates were obtained by calculating the percent damage in 1 mm increments through the anterior–posterior extent of V1 and V2 (4 sections, from −5.60 to −9.30 mm from bregma) [67]. Each section was assessed under magnification, and the tissue was considered damaged if it was absent or necrotic. The region damaged was drawn onto a control template for each section, and the area of damage was calculated using an automated tool in a computer graphics program (Canvas 8, Deneba). The area of damage was then summed across sections and calculated as the percentage of the total control V1 or V2 (V2MM+V2ML) areas respectively.

### Behavioral data analysis

Data analysis was performed with custom scripts and functions written in Matlab (Mathworks, Natick MA). To analyze performance by day (Figures 2, 3, and 5), we identified all trials performed on a given date, excluding trials that followed errors (as these are subject to bias due to correction trials, see above) to obtain the valid trials for each task. We excluded from analysis any days in which the rat's overall side bias in the valid trials was >15% (subject chose one of the two response ports >65% of trials), and required a minimum of 40 valid trials to analyze a task on a given day. Rats typically performed one or two tasks each day, and completed up to 2000 valid trials on a single task in a day (on average >500 valid trials per day per task analyzed). We report the end-of-day performance on each task, computed as the fraction correct on the last 100 valid trials on that task that day (or all the valid trials, if fewer than 100 performed).

In order to summarize the effects across the population (Figure 7), we define the pre-operative performance as the fraction correct on the last 100 valid trials for each task before surgery. If steady state performance was observed over multiple days for a task before surgery, we combined the last 100 trials from each day after reaching asymptotic performance on the task. To compute the post-operative performance we combined the last 100 trials from each day after performance reached steady state on the task, and report the fraction correct over this set of trials.
